# Behavior Emotion Therapy System and You: Co-Design and Evaluation of a Mental Health Chatbot and Digital Human for Mild to Moderate Anxiety in Healthy Participants

**DOI:** 10.2196/66163

**Published:** 2025-10-30

**Authors:** Almira Osmanovic Thunström, Lilas Ali, Hanne Krage Carlsen, Maria Bohm, Linda Wesén, Olof Wrede, Iris Sarajlic Vukovic, Andreas Hellström, Tomas Larson, Steinn Steingrimsson

**Affiliations:** 1 Institute of Neuroscience and Physiology, Gothenburg University, Journalvägen 5, Gothenburg, 41560, Sweden; 2 Psychiatric Department, Sahlgrenska University Hospital, Region Västra Götaland, Gothenburg, Sweden; 3 Centre of Registers Västra Götaland, Gothenburg, Sweden; 4 Crisis and Trauma Center, Regionshälsan, Region Västra Götaland, Gothenburg, Sweden; 5 Department of Technology Management and Economics, Chalmers University of Technology, Gothenburg, Sweden

**Keywords:** co-design, chatbot, mental health, digital human, prototyping, avatar, behavior emotion therapy system and you, BETSY, chatbots, conversational agent, digital human, anxiety, mixed-method, mixed-methods, social media, mental health support, self-help, healthcare, digital health

## Abstract

**Background:**

Co-design is a collaborative approach involving end users, stakeholders, and designers in creating digital tools for health care. This study focuses on the co-design and evaluation of Behavior, Emotion, Therapy System, and You (BETSY), a mental health chatbot and digital human for mild to moderate anxiety.

**Objective:**

This study aims to develop and evaluate BETSY through a co-design process involving potential users.

**Methods:**

The study used a mixed-methods approach across 3 phases. Phase 1 involved recruiting 87 volunteer participants through social media for initial end-user requirements. Phase 2 focused on the design process based on end-user requirements using the expertise of 10 stakeholders in health care and health service user. Phase 3 evaluated the 2 prototyped interfaces (a text-only chatbot and a voice-activated digital human chatbot) with 45 healthy volunteers.

**Results:**

For phase 1, 61% (n=51) of participants had previous experience with chatbots and 86% expressed a willingness or potential willingness to use a chatbot for mental health support. Thematic analysis in phase 2 revealed key user preferences for a personalized, nonjudgmental chatbot with a clear identity and a focus on empathy. Privacy concerns and the need for clear interaction guidelines were highlighted. In phase 3, the users showed a strong preference for discussing anxiety related to work, relationships, and health. Both text-only and voice-activated digital human users considered BETSY valuable for managing mild to moderate anxiety and providing self-help exercises.

**Conclusions:**

The co-design process yielded valuable insights for developing BETSY. While users recognized its potential as an accessible first step before seeking professional help, they also identified areas for improvement, including more nuanced conversation capabilities and a broader range of exercises. BETSY’s potential as a screening tool for health care was consistently acknowledged, suggesting its capability for alleviation of health care system burdens.

## Introduction

Co-design is a collaborative design approach that involves end users, stakeholders, and designers in the design process to create more effective and engaging digital tools. Co-design is an important aspect of creating apps and digital services in health care, as it ensures better design, overall outcome, and sustainability in projects aimed at reaching targeted audiences [1-5]. By involving end users in the design process, one is more likely to create design outcomes that create higher efficacy among its users, especially those who would benefit from continuous and stringent use of a digital tool, such as self-monitoring or digital therapy [1-3,5]. For instance, a study on codeveloping a mental health and well-being chatbot with and for young people demonstrated the benefits of involving end users in the design process [6]. The research used interviews and surveys to inform the creation of the chatbot’s personality and conversation design, resulting in a more effective and engaging tool for young adults. Another study of a co-design process for a chatbot service for mental health aimed at individuals living in rural areas showed that involving end users in the design process gave valuable insight into potential design, need, and architecture for a new mental health service [2,6]. The co-design process can be tailored according to the users, considering the diversity within the group, digital technology, and the setting. There are also potential challenges, particularly related to finding resources [3].

One of the core problems in design is ensuring representation. Lack of representation can lead to lower user efficacy and problems with an inclusive user interface. Co-design, an approach that involves the inclusion of potential users in the design process, can mitigate some of the issues of noninclusionary design and is a promising method for creating digital mental health tools that are more effective, engaging, and inclusive [4,5].

There are also matters of safety and trustworthiness. Co-design can be used to increase transparency as well as address concerns and potential hazards of the system before it is even developed [4]. A study by Hamlin et al [7] showed that skepticism toward digitalization decreased significantly when users were familiar with and felt in control of the safety and security of the digital health service provided to them.

By including experts who are also users of mental health services, it is possible to capture the perspectives of individuals who are vulnerable within the system [2,4,6]. However, there is still a standing stigma of involving patients and the general public as co-designers of mental health services [2], which has resulted in a lack of literature and results regarding co-design processes in chatbot-driven technologies and, in particular, in the design of digital humans. In a previous study [8], we detailed the history, usability, and needs for a mental health chatbot as well as providing a detailed description of the quantitative and biometric data used to evaluate a mental health chatbot and digital human interfaces for conversations on mental health. The overarching goal of this paper is to detail: (1) the co-design process of a mental health chatbot for mild to moderate anxiety and depression; (2) the design choices in the prototype of the chatbot and digital human system; and (3) qualitatively evaluate to what extent we had accurately captured the needs of the end users based on the co-design process in 2 different interfaces (text-based and voice-activated digital human). This was performed through 3 separate phases: inquiry, design, and evaluation.

## Methods

### Overview

**Figure 1.**
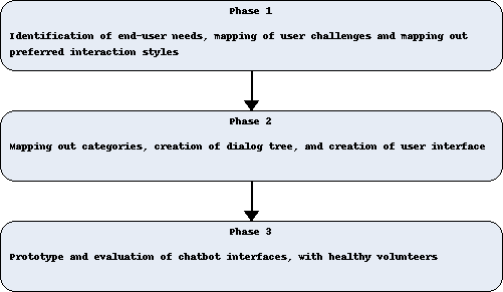
Phases of co-design development and evaluation.

This project comprised 3 phases. Phase 1 consisted of mapping end-user requirements, phase 2 focused on chatbot development based on end-user requirements, and phase 3 evaluated a chatbot prototype consisting of a chatbot prototype with 2 interface options (Figure 1). Throughout the project, healthy volunteers were engaged to ensure adherence to the regulatory and ethical standards governing the development of novel early-stage medical technologies.

### Phase 1

#### Data Collection and Design Framework for End User Needs

This phase of the project focused on exploring user-centered requirements for developing a text-based and digital human chatbot to alleviate symptoms of mild to moderate anxiety. The primary objective was to map the key topics, preferences, and concerns held by anonymous voluntary participants from the general public regarding a chatbot for mild to moderate anxiety. Ten experts were invited to participate in the construction of the foundation of the survey. The project team consisted of a lead engineer, 2 psychiatrists, 2 psychiatric nurses, 4 clinical psychologists, and 1 patient representative. There was an even representation of sex and age among the participants. Each member of the team was carefully selected based on their expertise and knowledge of digitalization and psychiatry. The patient representative was neither a former nor a current patient of any of the experts and was not under their care. The patient was seen as a valued and independent expert in the project. No monetary compensation was involved in participation by any of the experts and was voluntary for all parties. Together, the participants designed a survey to gather qualitative and quantitative data from voluntary participants regarding expectations for a chatbot designed to manage anxiety and their overall opinions on the use of technology for mental health interventions.

#### Survey Construction and Design

To gather both qualitative and quantitative data on user-centered requirements for developing a chatbot aimed to manage mild to moderate anxiety, a mixed-method survey was designed. Drawing on the efficiency, effectiveness, and satisfaction domains outlined by Radzwill and Benton [9], the survey explored participants’ perceptions and preferences regarding mental health chatbots. It included 8 multiple-choice and 3 open-ended questions, covering demographics, design expectations, functionality suggestions, and overall attitudes toward chatbot use in anxiety management.

The multiple-choice section addressed gender, education, and occupation, as well as preferences for interaction mode (text or digital human), chatbot appearance (human-like or avatar), content topics, level of personalization, and personality traits (eg, friendly vs neutral, personal vs clinical). The open-ended questions invited participants to reflect on their general attitudes toward mental health chatbots, perceived benefits and challenges in using them for anxiety support, and their views on chatbots as a complement or alternative to traditional care.

The open-ended questions were:

- What do you wish an anonymous conversation with a mental health chatbot would give you?
- What would make you hesitate to talk to a chatbot about mental health?
- Can you give an example of the worst and best chatbot you’ve ever talked to?

#### Collection of Survey Data and Analysis

The survey was distributed through social media networks such as Facebook (Meta), LinkedIn (Microsoft Corporation), Instagram (Meta), Twitter (X Corp), and Mastodon (Mastodon gGmbH) from April to June 2020. The collected data were analyzed using descriptive statistics and thematic analysis to identify patterns and insights related to the participants’ attitudes toward chatbots for mental health, particularly those tailored for anxiety management [9]. We implemented measures to safeguard the privacy and anonymity of participants. Data were collected through an anonymous, encrypted survey hosted on the free-to-use, noncommercial, platform PsyToolKit (owned by Professor Gijsbert Stoet). No personal identifiers or IP addresses were recorded before entering the survey, and a written statement was shown providing clear and concise information to participants about the study’s purpose, scope, and potential risks. Participants were given the opportunity to review and consent to participate before proceeding with the survey.

### Phase 2

#### Creating a Framework for Content and Interface

The second phase of the project aimed at translating the extracted themes and requirements into a preliminary structure for a dialog tree and avatar personality. This involved iterative and collaborative explorations of the chatbot’s backend, frontend, and aesthetics, including the design of the anthropomorphic features of the avatar. From July 2020 to December 2021, 4 workshops were conducted and iterative design work on the chatbot was carried out. These workshops consisted of invited experts listed previously and focused on exploring various aspects of chatbot technology, particularly interface and content design, which were guided by the insights gained from the phase 1 survey.

The expert participants were active in constructing the frame and content of the chatbot, not as testers of the prototype. To outline the chatbot functions, the experts were provided with Post-it Notes and instructed to adopt the perspective of a patient. They were then asked to formulate questions regarding their overall mental health, specifically focusing on mild to moderate anxiety and depression, with an emphasis on topics that received higher prioritization from the general public (Table 1). These questions were subsequently posted on a wall in a large conference room. Following this, the same 10 participants assumed the role of supportive mental health peers (not therapists) and responded to each other’s Post-it Notes. The collected questions and responses were used as a foundation for training the language model and identifying keywords for the rules-based chatbot in Google’s chatbot platform: DialogFlow.

**Table 1.** Phase 1: respondent demographics and responses to questions (N=87).

Demographic question and response	Respondents (N=87), n (%)	
Sex		
Male	29 (33)	
Female	54 (62)	
Other	2 (2)	
Rather not say	2 (2)	
Age (years)		
18‐25	3 (3)	
26‐35	15 (17)	
36‐45	37 (43)	
46‐55	26 (30)	
56+	5 (6)	
Do not want to answer	1 (1)	
Have you ever talked to a chatbot?		
Yes	51 (61)	
No	27 (32)	
I am not sure	6 (7)	
Would you talk to a chatbot anonymously about your mental health?		
Yes	31 (37)	
Maybe	41 (49)	
No	12 (14)	
If you would talk to a chatbot anonymously about your mental health, would you want it to have a name?		
Yes	46 (56)	
Maybe	19 (23)	
No	17 (21)	
If the chatbot had a name, would you want it to have a distinct identity (eg, calling itself a specific gender identity and with a human name?)		
Yes, I want a distinct identity	16 (20)	
I want it to be neutral, no specific identity	29 (36)	
I would not care	28 (34)	
I don’t know	8 (10)	
If you talked to a chatbot about your mental health, would you want it to be formal or informal when it addresses you?		
It should be completely informal like a friend	15 (18)	
It should be somewhat formal like an acquaintance	47 (58)	
It should be formal	19 (24)	
Which topic from this list would you like to include in the chatbot’s manuscript?		
Work	59 (68)	
Stress	58 (67)	
Relationships	56 (64)	
Disease	45 (52)	
Conflicts	40 (46)	
Family	38 (44)	
Partner	37 (42)	
Friendships	33 (38)	
Children	32 (37)	
Death	32 (37)	
Financial issues	31 (36)	
Abuse	29 (33)	
Medicine	29 (33)	
Exercise	29 (33)	
Food	26 (30)	
Addiction	23 (33)	
Legal issues	21 (24)	
Sex	20 (23)	
Sexuality	20 (23)	
Culture (movies, books, theater)	20 (23)	
Love	18 (21)	
Philosophy	18 (21)	
News	17 (19)	
Politics	15 (17)	
Art	12 (14)	
Weather	12 (14)	
Religion	10 (11)	
Fashion	7 (8)	
Celebrities	6 (7)	

To create the chatbot’s facial expressions and appearance, a standard model of face design from the New Zealand-based company UNEEQ (UneeQ Limited) was used. Since the survey participants from the general public did not express any specific preferences regarding gender, age, or other appearance features, the avatar was modeled after a nurse and given a female-presenting persona due to the statistical probability of meeting a female-presenting nurse in psychiatry in Sweden. To compare the effect of anthropomorphic features in a controlled manner, a text-only version of the algorithm was created using Itsalive.io (ItsAlive SARL) and published on a project-dedicated Facebook page. The model was named Behavior, Emotion, Therapy System, and You (BETSY).

### Phase 3

#### Evaluation of Chatbot Design

The final stage of the project aimed to assess the effectiveness of 2 prototypes: one based on text chat and the other based on interaction with a digital human avatar using only voice. As the comprehensive description of the procedures, scales, and questions used in the evaluation has been published previously by our group [8], the current paper will provide a summary of the overall methodology. Only the open-ended questions will be presented in this paper. In this manuscript, we focus on the qualitative evaluation of BETSY conducted in Phase 3, using open-ended questions analyzed through thematic analysis. An inductive, semantic coding approach was used to identify key patterns in user feedback, with themes interpreted in light of the design goals and user requirements established in phase 1. While this paper emphasizes qualitative findings, the companion publication [8] reports the full quantitative evaluation, including metrics such as the System Usability Scale (SUS) and biometric engagement indicators. To strengthen clarity and cross-reference between findings, we have added citations and brief summaries of those quantitative measures in the present manuscript, ensuring a more complete view of BETSY’s overall performance.

To ensure the inclusion of the target users, healthy individuals, advertisements were placed on major social media platforms to recruit volunteers for the evaluation of BETSY. For a more detailed description of the evaluation protocol, the reader is directed to our previous publication [8].

### Data Analysis

The study comprises a mixed methodology. For qualitative analyses, thematic analyses were explored through open-ended survey questions in phases 1 and 3 of this study. Thematic analysis is a widely used method for identifying, analyzing, and reporting patterns within qualitative data. This approach was chosen for its flexibility and ability to provide rich, detailed insights into the participants’ experiences and perspectives. An inductive approach was adopted, allowing themes to emerge from the data rather than fitting the data into a pre-existing coding frame. In addition, a semantic approach was used, focusing on the explicit content of the responses rather than underlying assumptions [10]. This methodological approach allowed for a systematic and rigorous exploration of participants’ perspectives, enabling the identification of key themes and patterns within the qualitative survey data.

To analyze the single- and multiple-choice questions answered by participants, we calculated and displayed the sums and percentages for each category. Since the goal of this study was not to compare groups, no statistical comparisons were conducted.

### Ethical Considerations

In phases 1 and 3 of the study, all participants provided written informed consent prior to participation. The study received approval from the national ethical committee (reference number DRN 2021‐02771 with amendment DRN 2024-03109-02). All procedures were conducted in accordance with the Principles of the Declaration of Helsinki (1975) and its subsequent revisions. All data collected were anonymized before analysis to protect participant privacy and confidentiality. The patient representative was actively involved in the project design and implementation. Their involvement was strictly voluntary and did not affect or interfere with their clinical care. No monetary or material compensation was provided for participation in this project.

## Results

### Phase 1

The phase 1 survey respondents (N=87) had little to no preference for the name and gender of the chatbot. The ideal chatbot persona should be personalized, akin to an acquaintance rather than a close friend. The survey revealed a strong willingness among participants to engage in anonymous conversations with chatbots about their mental well-being. Remarkably, nearly 7% of respondents were unsure whether they had interacted with a chatbot or a human. Work, stress, relationships, and conflicts emerged as the primary areas of interest for chatbot conversations (Table 1). While most participants found the provided topic list comprehensive, a few suggested the inclusion of “health stress.” As the participants contributed data anonymously, they are distinguished by letters in the quotes (Participant A, B, C, etc).

#### Lack of Human Connection

The qualitative data gathered from the phase 1 open-ended questions painted a nuanced picture of the participants’ attitudes toward chatbots for mental health support. While the potential benefits of anonymity, convenience, and availability were recognized, concerns about genuine human connection, empathy, and the effectiveness of chatbots were prevalent. Participants emphasized the importance of data security, trust, and ethical considerations in chatbot design, demanding clear guidelines and transparency regarding data handling. Participants expressed hesitations surrounding the limitations of chatbots in providing genuine human connection and empathy. They voiced concerns about the inability of chatbots to understand the depth and complexity of human emotions, particularly when dealing with sensitive mental health issues. The lack of personalized responses and the potential for generic advice further discouraged some participants.

*Because I don't believe it could empathize with me. Some subjects are fine, but just the fact that it’s not possible for it to relate to me, the fact being it’s a computer with programmed responses. Some subjects aren't really a possibility with a regular therapist however, and a chatbot could be useful to talk to about really embarrassing matters, assuming there wasn't a leak. There is definitely something to be gained there, but I wouldn't want to talk to a computer about my feelings, because it doesn't have any.*

[Phase 1: Participant A]

#### Privacy, Trust, and Data Security

Participants raised concerns about the privacy and security of their conversations with chatbots. They expressed uncertainty about how chatbot data are stored, who has access to it, and how it is used. The lack of clear guidelines and transparency regarding data handling eroded trust and discouraged open communication.

*I would hesitate due to security reasons. Where does the chat data end up when we are done. I also think I would be too much aware that I'm not talking to a human who can give me any kind of trust around where the data about the chats ends up. Talking to any human you might feel that this person will keep my secrets, but talking to a bot you know that the bot will do whatever it is programmed to do…*

[Phase 1: Participant C]

#### Utility and Effectiveness of Chatbots

Worries about the overall usefulness and effectiveness of chatbots for addressing mental health concerns were prevalent. Participants questioned the ability of chatbots to provide meaningful guidance and support, citing the lack of competence and the potential for misdiagnosis. They emphasized the importance of human interaction and expertise in providing comprehensive mental health care.

*Mental health issues need to be address by humans.*

[Phase 1: Participant E]

#### Design Requirements and Needs

Participants expressed clear requirements for a chatbot designed for mental health support. Users desired a chatbot that could provide a human connection and communication by offering reassurance, understanding, and empathy. In addition, users sought emotional support, guidance, and different perspectives on approaching challenges. They also wanted a chatbot that would validate their emotions and ensure they were not alone in their experiences. Finally, users valued features that facilitated self-care, personal growth, positivity, and motivation.

*Affirmation, if possible, or sense of understanding and that I'm not the only one with the issue.*

[Phase 1: Participant H]

*Reassurance that I will make it. Explain that the feelings I have are normal, many people have them without talking about it. And maybe just listen to what I say and hmm...*

[Phase 1: Participant I]

### Phase 2

**Figure 2.**
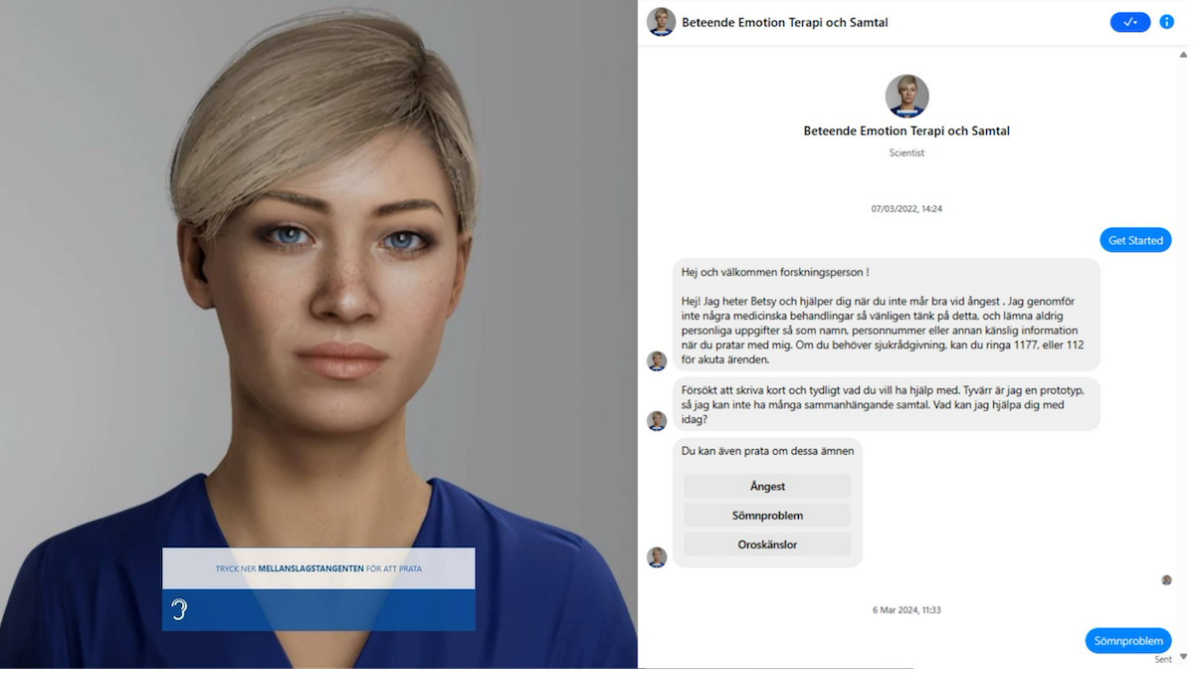
Two versions of Behavior, Emotion, Therapy System, and You: voice-activated digital human (left) and text-only chatbot (right).

In phase 2 of the study, design requirements were used as a guideline for content, personality, and BETSY’s appearance. The standard model offered by UNEEQ was used and resembled their previous avatar models (Figure 2). The group chose to extract a large portion of the topics from the survey in phase 1.

The chatbot’s topic selection process involved several key aspects, including relevance, specificity, user-centricity, comprehensiveness, balance, accessibility, user empowerment, collaborative approach, and continuous improvement. The topics focused on addressing the core aspects of anxiety and its triggers, while also providing personalized guidance and catering to the unique needs of each user. The chatbot covered a wide range of anxiety-related issues, offering a holistic approach to support and addressing the diverse spectrum of anxiety experiences. A careful balance was maintained between common and less-discussed anxiety triggers, ensuring that both widespread concerns and lesser-known aspects of anxiety management were addressed. The topics were designed to support users with knowledge and coping mechanisms, with the aim of enabling them to take an active role in managing their anxiety symptoms. To ensure that the tone of BETSY was in line with the group’s requirements for perceived empathy, the patient representative used BETSY at home continuously during the development process and reported back to the engineer; cases in which BETSY was perceived as less empathetic. This was not performed systematically or according to a scheme, as the patient representative experienced mental health problems and the team wanted to adapt the flow of the work as pragmatically as possible according to the patient representative’s capability at the time.

#### Dialog-Tree Development and Implementation

The experts started the dialog-tree development process by sorting and extracting keywords and phrases from the Post-it Notes produced from the workshop. Initially, the experts adopted a professional jargon-filled approach, posing technical challenges for the chatbot, such as repetitive answering of questions with subsequent questions. Recognizing this issue, the engineer provided guidance, encouraging the experts to adopt a nonclinical approach, responding as they would to a nonpatient user and adapting the language to include more everyday phrases without clinical jargon. This shift in approach resulted in more concise and targeted responses, enhancing the chatbot’s ability to provide effective guidance without venturing into diagnostics or triage. BETSY was provided with a soft-voice tone, slowing down the pace of her speech to decrease the probability of the avatar feeling stressed or risk making the user feel hurried in their interaction [11]. Concurrently, the experts collaborated on identifying and integrating relevant resources that could be linked alongside the chatbot’s responses. These resources were meticulously selected based on their credibility and authenticity, ensuring that only reliable and authoritative sources were provided (eg, the government site 1177.se). Links to governmental institutions and patient organizations were prioritized, guaranteeing that users had access to verified and reputable information. The compiled dialog tree, along with the training data comprising answered questions and related resources, was then integrated into 2 platforms: Dialogflow, a Google-based chatbot development platform, and Itsalive.io, a platform for text-based chatbots. The voice-based chatbot was released on a secluded page administered by Deloitte Digital (Deloitte Touche Tohmatsu Limited), while the text-based chatbot was deployed on Facebook from Meta on a secluded research page dedicated to BETSY. Users were to engage with both versions of BETSY on site (physical location) at Sahlgrenska University Hospital, maintaining their anonymity and without disclosing any personal information to the system.

To assess the performance of both chatbot versions, a test was conducted where both BETSY versions were asked the same question 100 times. In 99% of the cases, both chatbots provided identical responses. The only instance of discrepancy occurred when asked, “I have a hallucination. What should I do?” The digital human BETSY failed to comprehend the question, while the text-based BETSY accurately responded. The issue was fixed before participants were included in the study.

### Phase 3

A total of 45 healthy volunteers participated in the evaluation of the chatbot prototype (20 participants in the text-only group and 25 participants in the avatar group). The evaluation methodology has been published elsewhere by our group with a summary of the protocol and a complete table of demographic properties [8]. Based on the open-ended questions posed to the 45 participants regarding BETSY’s performance, there were positive and negative aspects to both interfaces (Table 2). As the participants were contributing data anonymously, they are referred to as numbers in the quotes to distinguish them (Participant 1, 2, 4, etc).

**Table 2.** Examples of positive and negative responses to Behavior Emotion Therapy System and You's text-only chatbot and voice-activated digital human.

Themes and question	Text-only	Voice-only
Usefulness		
Under what circumstances would you consider talking to BETSY^a^?	*For first contact. if I feel stressed, anxious, or have other simpler mental health problems, stress, screening.* [Participant 1]	*For lighter mental health condition. When I need quick and concrete help. BETSY provided clear and timely information, but didn’t seem to be that complex in her problem-handling skills.* [Participant 4]
—	*Practical exercises to deal with stress, muscle tension resulting from high workload.* [Participant 2]	*Concrete tips. The exercises were good. Being referred to info about my problem to learn more about different strategies was also good.* [Participant 5]
Screening	*For first contact if I feel stressed, anxious, or have other simpler mental health problems.* [Participant 6]	*Before I contact my healthcare provider.* [Participant 7]
Taking offense		
Is there anything that BETSY said that you would take offense to hearing when you felt bad?	*Repetitive answers that are not grounded in what we talked about...I feel unseen and unheard.* [Participant 8]	*I probably would have reacted to the tone of voice and the number of words. A real person would talk less and listen more, at least until the problem was circled. I had reacted to words she emphasizes wrong. It becomes clear that it is a robot. Her face and eyes feel more person-like, alive.* [Participant 9]
Emotional connection		
Did you feel any connection to BETSY?	*No, not so much. Probably because of the repetitiveness. Felt like I’m not always heard*. [Participant 10]	*Yes, I found her gaze present. The way she talked, a soft and pleasant voice. Clear when she made claims/advice.* [Participant 11]
Negative technical aspects		
What would you like to improve with BETSY?	*More interactivity, not so much repetition of the same response. If I’ve already received sleep tips/link, I don’t want the same more times. She should understand better what I’m asking of her.* [Participant 12]	*The exchange became ’choppy’, felt that BETSY did not want to listen to how I described my feelings. The answers/feedback were related directly to the facts. Lacked the sense of empathy.* [Participant 13]
Overall positive aspects		
Did you feel any connection to BETSY?	*Above all, that the answers felt more personal and not just fact-based text but a more personal language.* [Participant 14]	*Yes, she had a pleasant voice and left a pleasant impression.* [Participant 15]
Suggestions for the future		
What would you like to improve with BETSY?	*Possibly her ability to pick things up out of the conversation. More nuanced. Clearly, she picked up guiding words in the sentence and not sentences, which sometimes made the support incorrect.* [Participant 16]	*Voice understanding. Not linking out to YouTube. Feels frivolous, the logic and understanding that I was talking about. She needs to get better at keeping the conversation going*. [Participant 17]

a BETSY: Behavior, Emotion, Therapy System, and You.

Users of both interfaces considered BETSY a valuable adjunctive tool for the management of mild to moderate anxiety and for providing self-help exercises. Participants emphasized the need for a more comprehensive set of exercises, particularly for stress management and focus enhancement. BETSY’s potential as a screening tool for health care was consistently recognized. Users viewed BETSY as an accessible first step before seeking professional help. The suggestion to integrate a screening procedure into BETSY’s capabilities highlights its potential to alleviate health care system burdens.

Despite BETSY’s perceived usefulness, participants identified conversational issues. Both groups found BETSY overly talkative and her responses excessively lengthy. The tendency to divert conversations from relevant topics raised concerns about being unheard and unseen by several users. These issues emphasized the need for more responsive and relevant conversational design. Some participants, particularly in the digital human group, reported experiencing an emotional connection with BETSY, attributing it to her sympathetic, kind, and comforting nature. This finding underscored the potential for chatbots to establish meaningful interactions, especially in voice-activated interfaces. Further research could explore methods to enhance this connection for a wider user base. Most participants expressed concerns about BETSY’s technical aspects, viewing it as incomplete. Issues included repetitive responses, misinterpretations of user input, button visibility in the voice-activated digital human interface, and looping in the text-only version. The most disruptive aspect was redirecting users to external webpages for relaxation exercises and information. Participants suggested presenting information within the chat interface for seamless conversational flow.

## Discussion

### Principal Findings

In this study, we aimed to co-design a conversational automated interface and system intended to alleviate mild to moderate anxiety symptoms. Through a 3-phase process, we explored end-user requirements by integrating end-user input from the general public. Using the end-user requirements together with specialized expertise, 2 interfaces were developed of the BETSY chatbot: a text-based and a digital human voice-activated avatar. Due to regulatory restrictions surrounding medical devices and technology targeting mental health issues, we enlisted the assistance of healthy participants to assess the system’s usability and its alignment with the initial end-user survey’s requirements, while at the same time consistently keeping a user-centered perspective through the involvement of a patient representative as a continuous source for testing and validation. Similar to prior research, end users identified privacy and security as critical factors influencing their trust in a potential system [7]. Work and relationships emerged as highly ranked topics that end users desired to discuss with a potential system. While we only received responses from 87 members of the general public, we observed a diverse range of perspectives and interests, which we hypothesize accurately reflects the general population’s fears, concerns, and interests: this has been shown to be frequently present in other studies [12]. However, the survey’s exclusive distribution via social media may have excluded individuals without social media accounts, potentially limiting the diversity of perspectives. Due to the anonymous nature of the phase 1 survey where metadata such as IP addresses was omitted, it is not possible to decipher whether one person had answered the survey multiple times. Despite this limitation, we noted that phase 1 respondents expressed diverse perspectives and different levels of enthusiasm for chatbot technology, including individuals who were skeptical or unfamiliar with the technology, which suggests a broad representation of the general public. One recurring concern was the system’s inability to understand feelings and relation to human emotions, which could hinder feelings of usability and connection to the chatbot. This has been observed in other studies in which the participants felt hesitations about sharing emotional states with a system that could not understand what it felt like to feel, thus could not produce true empathy [12,13]. It is also worth noting that this examination was performed before the release of commercial large language models (LLMs) or systems which use these for therapy, such as Character.ai (Character Technologies, Inc), OpenAI (OpenAI, Inc), etc; thus, the opinions of the participants do not reflect those systems but are solely focused on rules-based neuro-linguistic programming (NLP) systems with little or no machine learning capability. Conversations that are constructed using LLMs are less likely to produce the same level of readiness and repetitiveness as rules-based NLP models such as BETSY [14,15]. Many, if not all problems of repetitiveness are minimized if not diminished through the use of LLMs.

A personalized, nonjudgmental chatbot with a clear identity (named) and a focus on empathy was preferred by participants. While demographic data were collected in phase 1 and used to guide certain design decisions, we recognize the value of making these connections more explicit. Participant preferences for a semiformal tone, for example, directly informed BETSY’s conversational style, with the majority favoring communication “like an acquaintance” (58%). Similarly, the decision to implement a female-presenting avatar was influenced by local psychiatric care demographics, where female nurses are more prevalent. While these choices were rooted in user feedback and contextual relevance, the sample’s representativeness was limited by the recruitment strategy, which primarily relied on social media platforms and lacked detailed data on ethnicity or socioeconomic background. Future design phases will aim for broader inclusion to explore how a more diverse demographic composition might inform personalization and user experience.

A strong willingness to engage anonymously with chatbots for mental well-being was identified. However, concerns regarding data security and privacy need to be addressed to build user trust. Attitudes toward digital care change when trust in cybersecurity is adequate. This was evident in both our investigation and investigations of others [7,12,13].

In phase 2, we used the end-user requirements as a guiding principle for the prototype, incorporating as many features as possible within the prototype’s framework. We aimed for a representative and equitable group of experts in terms of gender, age, and areas of expertise. The system was designed as a female avatar, a choice based on gender representation statistics from our hospital. However, in retrospect, given the lack of preferences expressed by phase 1 respondents, we could have explored more diverse options. Female-presenting avatars are commonly used in research on digital humans [16]. Exploring alternative representations could have provided insights beyond those currently available in other studies. In future phases, we plan to diversify the representations available to end users. Diversity represented in avatars can help to broaden the trustworthiness and representations of the mental health profession [17], especially as in our broader study, we found that women were more prone to report irritation when talking to BETSY [18].

Our iterative design process yielded valuable insights. Regrettably, due to the scope of this investigation, we did not collect our own thoughts and experiences in a qualitative or quantitative manner. We believe that including expert reflections and evaluations would have enriched the results of this co-design initiative. In future studies, we will incorporate these considerations. We chose to explore 2 interfaces, text-only and voice paired with a face with anthropomorphic features. Face-to-face communication is unique and adds many dimensions to communication, both in human animals and others. Unlike interacting with objects, faces trigger a sense of ethical responsibility [18]. Systematic evaluations of chatbot research have shown that the level of humanness is a key factor in the usability of chatbots [19]. In phase 2, we explored incorporating expressions that leaned toward empathetic and semiformal. In our efforts to create equally empathetic interfaces, we explored research on text chatbots, which yielded a comprehensive list of traits that were desirable, such as affirmative language, ability to show affect toward the subject, and a high level of consciousness [11,12,20-25]. In 2002, Morris [26] proposed that using a language which corresponds with the emotional state of the chatbot (eg, in the case of BETSY, showing irritation or anger toward a workplace bully when the user notifies her that they have been subjected to such behavior) along with their given role (eg, caregiver) and social awareness (comforting a person in distress) results in more believable interactions [26].

### Comparison With Previous Work

In our evaluation of BETSY, we employed healthy volunteers (for more detailed methods see [8]). Our evaluation of the solution indicated that most end-user requirements were met. The system was perceived as useful both in terms of qualitative markers as shown in this co-design evaluation as well as quantitatively through the System Usability Scale in our previous publication of the quantitative metrics related to this investigation [8]. Similar to the end-user requirements noted in phase 1 of the co-design of the prototype, the participants who evaluated the prototype noted that BETSY was useful in providing affirmation and information about specific mental health problems and about the mental state they were either experiencing or simulating to BETSY, that is, stress, problems at work, and more. Our design choice yielded a positive response with regard to her persona, voice, and feeling of presence in the digital human version of BETSY, and the results correspond to other studies in the field [11]. Overall, the design of BETSY was well received and highlighted, as other studies have proposed [2-5,11], that working together with end users before initiating the design process is an important component in creating user-friendly experiences, independently of which interface is targeted for the chatbot.

### Limitations

Several technical challenges and limitations identified in this investigation could be addressed through the usage of LLMs or other systems capable of generating responses based on instructions and datasets containing sufficient information for generating novel and relevant responses [14]. However, these technologies were not available at the inception of this project and were thus not used in the design. In addition, while LLMs have demonstrated high usability and emotional connectivity in some studies [14], they can also exhibit unpredictable behavior and pose risks to vulnerable individuals through the generation of harmful responses such as hallucinations [27,28] or the potential compromise of personal data in systems owned by private entities that provide limited transparency regarding their LLM-training methodologies. As BETSY was never used outside the scope of the tests of this prototype, we acknowledge that some of the concerns and aspects of potential chatbot systems were not addressed, such as cybersecurity. When creating a system that handles something as private and vulnerable as an individual’s mental health, it is of utmost importance to discuss the ethical and legal implications of the digital infrastructure of the systems, as there are many aspects of technology that might lead to harm for the user [28,29]. In our study, we opted to protect users by providing them with an on-site computer and instructing them to avoid personal problems. This would be neither feasible nor probable in the delivery of a real system, a system which would be dependent on strong end-to-end encryption, possible autodeletion of messages, and careful IT architecture in order to address the safety and security concerns from phase 1 end-user requirements. As deployment of a mental health service was not the aim of this study, we did not delve into potential digital infrastructure. In future studies and product development, we will make the results of this co-design initiative central to our work as well as to explore, test, and develop methodologies that enable safe and ethical user experience [30].

### Conclusions

We have developed a conversational automated interface and system, BETSY, by co-designing with end users and experts. Collaboration and co-design resulted in a system that was positively received by healthy volunteers. The system met most end-user requirements and was perceived as useful. While LLMs could address some technical limitations and negative technical aspects of BETSY (eg, repetitiveness), the potential risks and ethical considerations warrant further evaluation before their incorporation into mental health-related systems.

## Abbreviations

BETSY: Behavior, Emotion, Therapy System, and You
LLM: large language model
NLP: neuro-linguistic programming

## References

[1] Bevan Jones R, Stallard P, Agha SS (2020). Practitioner review: co-design of digital mental health technologies with children and young people. J Child Psychol Psychiatry.

[2] Wright M, Getta AD, Green AO (2021). Co-designing health service evaluation tools that foreground first nation worldviews for better mental health and wellbeing outcomes. Int J Environ Res Public Health.

[3] Potts C, Ennis E, Bond RB (2021). Chatbots to support mental wellbeing of people living in rural areas: can user groups contribute to co-design?. J Technol Behav Sci.

[4] Patrickson B, Musker M, Thorpe D, van Kasteren Y, Bidargaddi N, The Consumer and Carer Advisory Group (CCAG) (2023). In-depth co-design of mental health monitoring technologies by people with lived experience. Future Internet.

[5] Porche MV, Folk JB, Tolou-Shams M, Fortuna LR (2022). Researchers’ perspectives on digital mental health intervention co-design with marginalized community stakeholder youth and families. Front Psychiatry.

[6] Grové C (2020). Co-developing a mental health and wellbeing chatbot with and for young people. Front Psychiatry.

[7] Hamlin M, Steingrimsson S, Cohen I, Bero V, Bar-Tl A, Adini B (2020). Attitudes of the public to receiving medical care during emergencies through remote physician-patient communications. Int J Environ Res Public Health.

[8] Thunström AO, Carlsen HK, Ali L, Larson T, Hellström A, Steingrimsson S (2024). Usability comparison among healthy participants of an anthropomorphic digital human and a text-based chatbot as a responder to questions on mental health: randomized controlled trial. JMIR Hum Factors.

[9] Radziwill NM, Benton MC (2017). Evaluating quality of chatbots and intelligent conversational agents. arXiv.

[10] Naeem M, Ozuem W, Howell K, Ranfagni S (2023). A step-by-step process of thematic analysis to develop a conceptual model in qualitative research. Int J Qual Methods.

[11] Chaves AP, Gerosa MA (2021). How should my chatbot interact? A survey on social characteristics in human–chatbot interaction design. Int J Hum Comput Interact.

[12] Abd-Alrazaq AA, Alajlani M, Ali N, Denecke K, Bewick BM, Househ M (2021). Perceptions and opinions of patients about mental health chatbots: scoping review. J Med Internet Res.

[13] Chin H, Song H, Baek G (2023). The potential of chatbots for emotional support and promoting mental well-being in different cultures: mixed methods study. J Med Internet Res.

[14] Martins A, Londral A, L Nunes I, V Lapão L (2024). Unlocking human-like conversations: scoping review of automation techniques for personalized healthcare interventions using conversational agents. Int J Med Inform.

[15] Dale R (2021). GPT-3: what’s it good for?. Nat Lang Eng.

[16] Wilson L, Marasoiu M (2022). The development and use of chatbots in public health: scoping review. JMIR Hum Factors.

[17] Do TD, Zelenty S, Gonzalez-Franco M, McMahan RP (2023). VALID: a perceptually validated Virtual Avatar Library for Inclusion and Diversity. Front Virtual Real.

[18] Corti K, Gillespie A (2015). A truly human interface: interacting face-to-face with someone whose words are determined by a computer program. Front Psychol.

[19] Rapp A, Curti L, Boldi A (2021). The human side of human-chatbot interaction: a systematic literature review of ten years of research on text-based chatbots. Int J Hum Comput Stud.

[20] Shum H yeung, He X dong, Li D (2018). From Eliza to XiaoIce: challenges and opportunities with social chatbots. Frontiers Inf Technol Electronic Eng.

[21] Croes EAJ, van Wezel MMC, Antheunis ML I’m here for you": can social chatbots truly support their users? A literature review.

[22] Vaidyam AN, Wisniewski H, Halamka JD, Kashavan MS, Torous JB (2019). Chatbots and conversational agents in mental health: a review of the psychiatric landscape. Can J Psychiatry.

[23] Ruane E, Farrell S, Ventresque A User perception of text-based chatbot personality.

[24] Kang M (2021). Non-verbal emotional expressions for social presence of chatbot interface. J Korea Contents Assoc.

[25] Denecke K, Vaaheesan S, Arulnathan A (2020). A mental health chatbot for regulating emotions (SERMO) - concept and usability test. IEEE Trans Emerg Topics Comput.

[26] Morris TW Conversational agents for game-like virtual environments. https://aaai.org/papers/0016-SS02-01-016-conversational-agents-for-game-like-virtual-environments/.

[27] Alkaissi H, McFarlane SI (2023). Artificial hallucinations in ChatGPT: implications in scientific writing. Cureus.

[28] Blease C, Torous J (2023). ChatGPT and mental healthcare: balancing benefits with risks of harms. BMJ Ment Health.

[29] Vilaza GN, McCashin D (2021). Is the automation of digital mental health ethical? Applying an ethical framework to chatbots for cognitive behaviour therapy. Front Digit Health.

[30] Banerjee S, Agarwal A, Bar AK (2024). Securing well-being: exploring security protocols and mitigating risks in AI-driven mental health chatbots for employees. Am J Comput Sci Technol.

